# P-1639. Platelet-Rich Plasma Therapy for Resistant Post-COVID-19 Olfactory Dysfunction: A Systematic Review and Meta-Analysis of Placebo-Controlled Studies

**DOI:** 10.1093/ofid/ofaf695.1815

**Published:** 2026-01-11

**Authors:** Mohamed Hamouda Elkasaby, Ahmed Elkoumi, Omar Elkoumi

**Affiliations:** Faculty of Medicine, Al-Azhar University, Cairo, Egypt., Cairo, Al Qahirah, Egypt; Faculty of Oral and Dental Medicine, Egyptian Russian University, Badr City, Al Qahirah, Egypt; Faculty of Medicine, Suez University, Suez, Egypt, Suez, As Suways, Egypt

## Abstract

**Background:**

Persistent olfactory dysfunction (OD) following COVID-19 remains a significant health concern. This study evaluates platelet-rich plasma (PRP) as a treatment for chronic, treatment-resistant post-COVID-19 OD.

**Figure:**
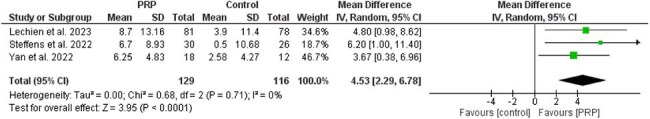
Forest plot of total TDI score at the longest follow up

**Figure:**
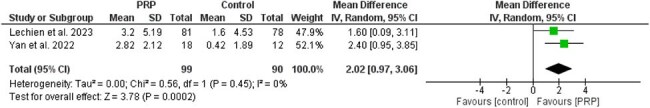
Forest plot of discrimination

**Methods:**

We searched PubMed, Scopus, Web of Science, Embase, and Cochrane databases through November 28, 2024, for studies on patients with post-COVID-19 OD unresponsive to standard treatments, treated with PRP injections into the olfactory clefts compared to placebo. The main outcome was the change in Sniffin’ Sticks TDI (threshold, discrimination, identification) scores. Pooled mean differences (MD) and 95% confidence intervals (CI) were calculated using a random-effects model.

**Results:**

Five studies including 330 patients were analyzed, with 171 in the PRP group and 159 in the placebo group. PRP significantly improved total TDI scores over placebo (MD = 4.53, 95% CI: [2.29, 6.78], P < 0.001) and specifically improved taste discrimination (MD = 2.02, 95% CI: [0.97, 3.06], P < 0.001). However, changes in taste threshold and identification were not significant (P = 0.28 and P = 0.14, respectively).

**Conclusion:**

PRP significantly enhances TDI scores in chronic post-COVID-19 OD patients, highlighting its potential as a therapeutic option for this challenging condition.

**Disclosures:**

All Authors: No reported disclosures

